# The roles of microRNAs in horticultural plant disease resistance

**DOI:** 10.3389/fgene.2023.1137471

**Published:** 2023-02-27

**Authors:** Aiai Zhang, Shunshun Zhang, Feng Wang, Xianmin Meng, Yue Ma, Jiantao Guan, Feng Zhang

**Affiliations:** ^1^ State Key Laboratory of Vegetable Biobreeding, Institute of Vegetables and Flowers, Chinese Academy of Agricultural Sciences, Beijing, China; ^2^ College of Plant Protection, Shenyang Agricultural University, Shenyang, China; ^3^ College of Horticulture, Shenyang Agricultural University, Shenyang, China

**Keywords:** disease resistance, microRNA, horticultural plants, plant defense, gene expression

## Abstract

The development of the horticultural industry is largely limited by disease and excessive pesticide application. MicroRNAs constitute a major portion of the transcriptomes of eukaryotes. Various microRNAs have been recognized as important regulators of the expression of genes involved in essential biological processes throughout the whole life cycle of plants. Recently, small RNA sequencing has been applied to study gene regulation in horticultural plants. In this review, we summarize the current understanding of the biogenesis and contributions of microRNAs in horticultural plant disease resistance. These microRNAs may potentially be used as genetic resources for improving disease resistance and for molecular breeding. The challenges in understanding horticultural plant microRNA biology and the possibilities to make better use of these horticultural plant gene resources in the future are discussed in this review.

## Introduction

MicroRNAs are a class of non-coding small-molecule RNAs in eukaryotes, mostly 21–24 nt in length, whose precursors are specific RNA sequences with hairpin structures (Voinnet, 2009). MicroRNAs regulate target genes through transcript cleavage or translational repression at the post-transcriptional level. Since the first report of plant microRNAs in Arabidopsis in 2002 (Llave et al., 2002), many studies have shown that microRNAs play vital roles in regulating biotic and abiotic stress conditions. MicroRNAs act as environmental response factors, inducing plants to overexpress or downregulate certain microRNAs or synthesize new miRNAs in response to stresses, promoting plant evolution and adaptation.

Genome-based microRNA breeding has been applied to many horticultural plants, including Solanaceae, Cucurbitaceae, and Cruciferae, thereby facilitating molecular breeding at the single-nucleotide level. In the last few years, microRNAs have been shown to play vital roles in various biological processes related to cell growth and differentiation, as well as the regulation of immune responses and agronomic traits. Therefore, in this review, we collected horticultural plant microRNAs through the sRNAanno database (Chen et al., 2021) to summarize the functions of microRNAs and the key roles of their target genes in disease resistance.

In the following section, we summarize recent findings and current progresses in the involvement and roles of microRNAs in horticultural plant disease resistance. To date, a variety of microRNAs have been identified in various plants and other organisms that play roles in regulating disease resistance signaling pathways, including disease-resistance-related gene expression, hormone signaling, and reactive oxygen species (ROS) production. We then propose research perspectives in microRNA-based technologies in horticultural plant disease control.

## The biogenesis of microRNAs: Biogenesis and mechanism of gene regulation

MicroRNAs are transcribed by the action of RNA polymerase II or III to produce the microRNA primary transcript (pri-microRNA) (Bologna and Voinnet, 2014). pri-microRNA is then 5′-capped and 3′-polyadenylated. Depending on its stem–loop structure, pri-microRNA is sequentially sliced by an RNase III family enzyme DICER-LIKE1 (DCL1), forming the microRNA/microRNA* duplex. During microRNA processing, DCL1 forms a complex with other RNA-binding proteins such as HYPONASTIC LEAVES 1(HYL1), SERRATE (SE), TOUGH (TGH), and DA/WDLE (DLL) (Borges and Martienssen, 2015). After the action of DCL enzymes, microRNAs undergo specific modifications that affect their stability. The duplex is 2′-O-methylated at the 3′ end by the methyl transferase HUA ENHANCER1 (HEN1) to prevent degradation (Modepalli et al., 2018). Then, pri-microRNA is exported from the nucleus to the cytoplasm *via* the HASTY (HST) transporter (Park et al., 2005). In Arabidopsis, HST is likely associated with the formation of the microRNA biogenesis complex at microRNA genes, promoting the transcription and processing of pri-microRNA rather than the direct export of processed microRNA from the nucleus (Cambiagno et al., 2021).

**FIGURE 1:**
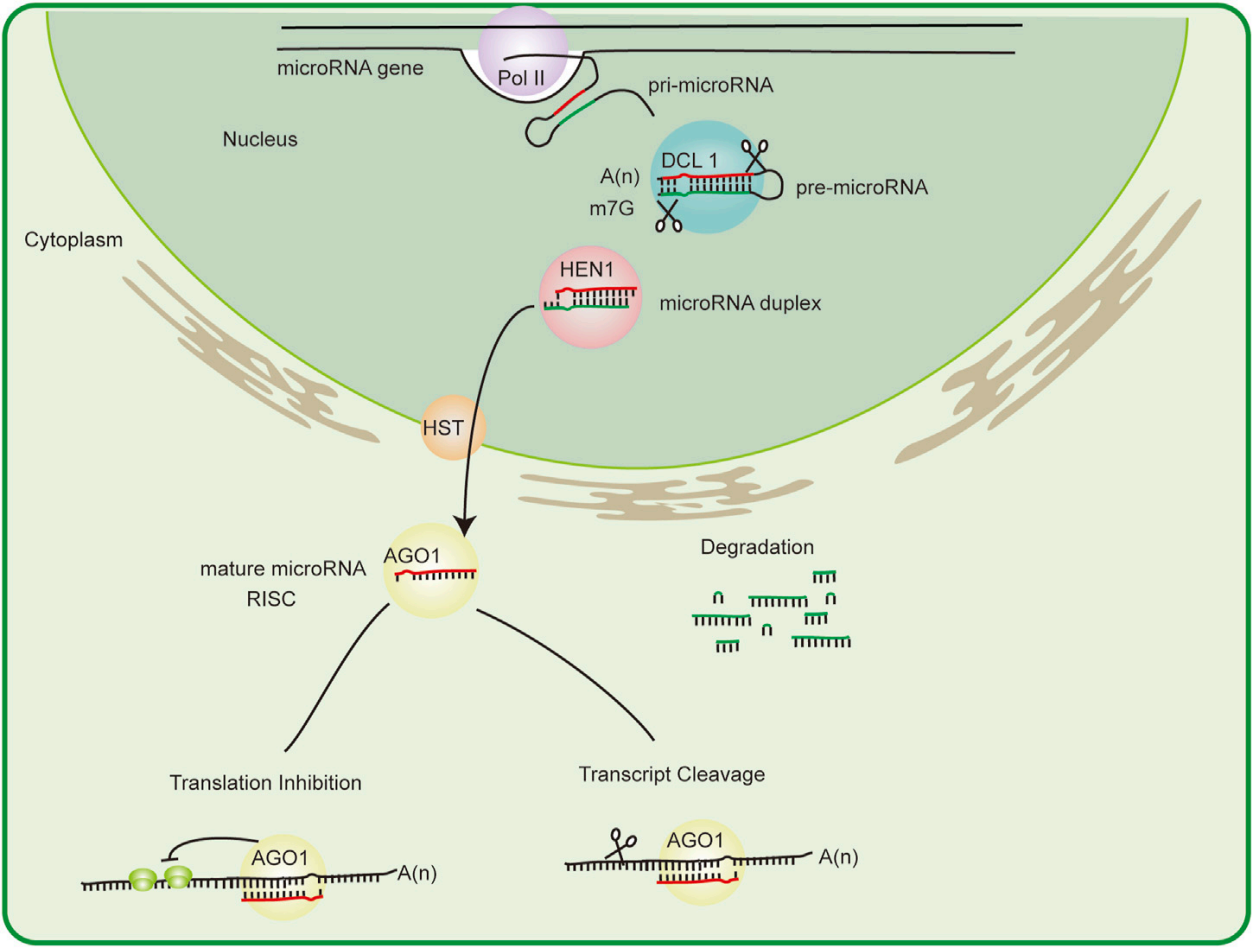
MicroRNA biogenesis and mechanism. The pathway of microRNA biogenesis in plants is shown. pri-microRNA is the primary transcript. The stem–loop structure of pri-microRNA is split in the nucleus by DCL1 to form a mature transcript. A red strand (microRNA) and a green strand (microRNA*) are generated. MicroRNA is stabilized by HEN1 methylation before being exported to the cytoplasm by HST. The red strand is integrated into miRISC, and the green strand is degraded. Depending on the degree of complementarity with the target site, miRISC cleaves mRNA, thereby inducing immediate degradation or inhibiting the translation process. AGO, Argonaute; DCL, Dicer-like protein; HEN1, HUA ENHANCER1; HST, HASTY; pri-microRNA, primary microRNA.

In the cytoplasm, the mature microRNA strand is transferred into Argonaute 1 (AGO1) protein to form the RNA-induced silencing complex (RISC), which eventually generates a functional single-stranded microRNA. Also, the other microRNA strand degrades rapidly. Plant microRNAs regulate target genes at the post-transcriptional level using both transcript cleavage and translational repression mechanisms. RISC can specifically recognize targeted mRNA degradation- or translation inhibition-mediated gene silencing *via* the Watson–Crick complementarity principle (Yu et al., 2017) (Figure 1).

## Horticultural plant microRNAs are highly diversified

**FIGURE 2:**
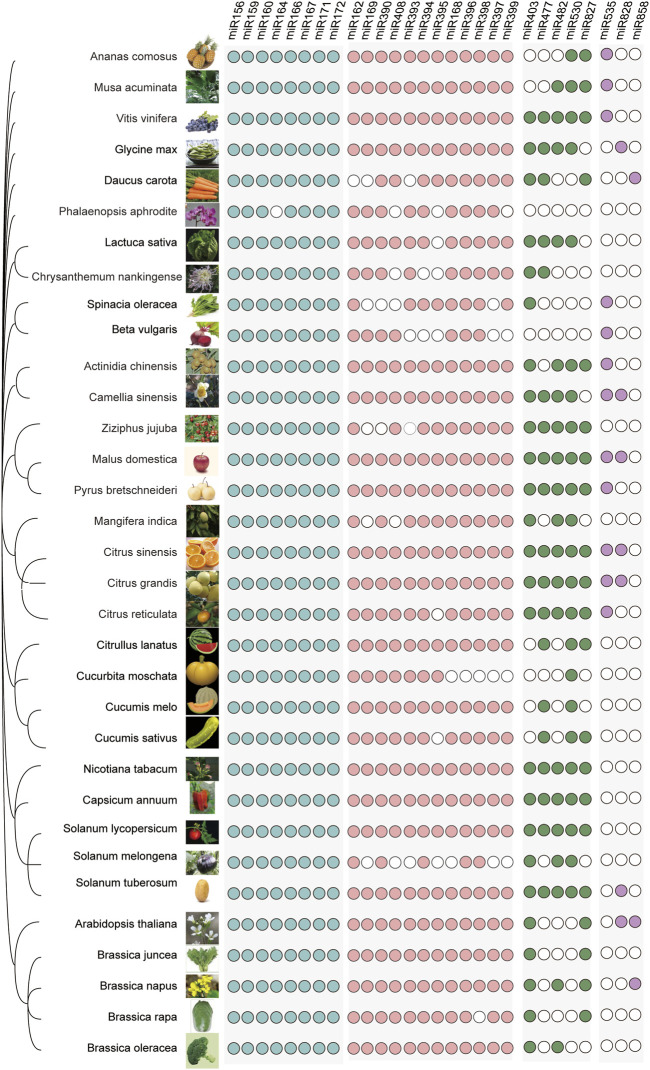
Conservation of miRNAs of horticultural plants. A phylogenetic tree of representative horticultural plants is shown on the left; major groups in the top left are the different families of horticultural plants: Asteraceae, Fabaceae, Apiaceae, Chenopodiaceae, Vitaceae, Musaceae, Bromeliaceae, Rhamnaceae, Rosaceae, Actinidiaceae, Rutaceae, Theaceae, Orchidaceae, Cucurbitaceae, Solanaceae, and Brassicaceae. Different miRNAs are listed at the top, grouped by conservativeness. Below the miRNAs, the colored circles indicate the presence of the component, while empty circles indicate the absence of the component.

With the deepening of the research on microRNAs in horticultural plants, more and more sources of evidence show that microRNA plays an important role in regulating many aspects of the growth and development of horticultural plants. To focus on microRNAs, we downloaded all the miRNAs of 33 phylogenetically representative horticultural plants (Figure 2) from the sRNAanno database, including 19 types of vegetables, 11 types of fruit trees, and three types of ornamental plants. These horticultural plants belong to the following families: Asteraceae, Fabaceae, Apiaceae, Chenopodiaceae, Cucurbitaceae, Solanaceae, Brassicaceae, Vitaceae, Musaceae, Bromeliaceae, Rhamnaceae, Rosaceae, Actinidiaceae, Rutaceae, Theaceae, and Orchidaceae. We collected 28 miRNA families to illustrate their conservation. In vegetables, we counted 23, 25, 21, 18, 16, 23, 16, 22, 22, 25, 16, 26, 25, 22, 24, 21, and 22 microRNA families in *Lactuca sativa*, *Glycine max*, *Daucus carota*, *Spinacia oleracea*, *Beta vulgaris*, *Citrullus lanatus*, *Cucurbita moschata*, *Cucumis melo*, *Cucumis sativus*, *Capsicum annuum*, *Solanum melongena*, *Solanum tuberosum L*., *Solanum lycopersicum L*., *Brassica juncea*, *Brassica napus*, *Brassica rapa*, and *Brassica oleracea*, respectively. In these vegetable crops, we found eight highly conserved microRNAs: miR156, miR159, miR160, miR164, miR166, miR167, miR171, and miR172. Among fruit trees, *Malus domestica*, *Citrus sinensis*, and *Citrus grandis* lack only the miR858 family, while *Pyrus bretschneideri* and *Vitis vinifera* lack not only the miR858 family but also the miRNA828 family. A total of 16 miRNA families are highly conserved in fruit trees, and 15 microRNA families are highly conserved in ornamental plants. We counted 19, 26, and 16 microRNA families in *Chrysanthemum nankingense*, *Camellia sinensis*, and *Phalaenopsis Aphrodite*. Highly conserved microRNAs often play an important role in plant growth and development. In four genera of Cucurbitaceae, we identified 23 microRNA families, 15 (65%) of which were found in all four genera. Within the Solanaceae, we identified 26 microRNA families, of which only eight (31%) were present in *S. melongena*. In five genera of the Brassicaceae family, we identified 25 microRNA families, of which 20 (80%) were present in all five genera. It is worth noting that miR535 is absent in other vegetable crops but present in Chenopodiaceae including *S. oleracea* and *B. vulgaris*.

## MicroRNAs regulate the expression of disease-resistance-related genes

There are a variety of pathogens that cause plant diseases, and the main ones in horticultural plants are bacterial, fungal and viral pathogens. Many microRNAs exhibit complex expression patterns and often regulate a series of biological processes through microRNA–target gene interactions, including development, signal transduction, environmental stresses, and host–pathogen interactions (Zhai et al., 2011). MicroRNAs are involved in the pattern (PAMP)-triggered immunity (PTI) and second-layer effector-triggered immunity (ETI) pathways and regulate disease resistance genes directly and indirectly (Zhang et al., 2019). Through the existing research, it was found that miR482/miR2118 responds to fungal infections caused by *Phytophthora infestans* and *Fusarium oxysporum* in tomatoes by regulating the downstream target gene *NBS* (Shivaprasad et al., 2012; Ji et al., 2018; Jiang et al., 2020; Hong et al., 2021). In apples, a 22-nt microRNA named miRcand137 compromises host resistance to *Botryosphaeria dothidea* infection. miRcand137 directs the silencing of *ERF14* that codes a transcription activator of several *PR* genes (Yu et al., 2022). In addition to fungal diseases caused by oomycete pathogens such as *Fusarium oxysporum* and *Phytophthora infestans* in tomatoes (Ouyang et al., 2014; Jiang et al., 2018; Canto-Pastor et al., 2019) and *Plasmodiophora* in *B. rapa* (Paul et al., 2021), miR482-NBS modulates resistance to bacterial diseases. Meanwhile, miR482-NBS plays an important role in regulating resistance to vegetable viral diseases caused by *cucumber mosaic virus* (CMV) in tomatoes (Feng et al., 2014). The genes of the serine/threonine protein kinase (STK) family play a key role not only in adaptation to abiotic stresses but also in activating plant defense mechanisms (Afzal et al., 2008). Cheng et al. (2016) found that the application of exogenous ABA resulted in multiple microRNAs targeting pathogen resistance genes, with miR319 targeting STK, which belongs to the STK family, being elevated. These receptors trigger signal transduction cascades, leading to rapid defense responses, hypersensitivity reactions, and programmed cell death to limit pathogen proliferation. Overall, these findings suggest that microRNA-mediated gene silencing might act as a key regulator for R gene-mediated defense responses.

## MicroRNAs are involved in plant hormone signaling

Plant hormones like ethylene, salicylic acid (SA), and jasmonic acid (JA) can act as signaling molecules involved in plant immunity, and microRNAs play a regulatory role in the signaling of these hormones (Dong et al., 2023). ARF acts as defense response transcription factors (TFs) that interact with cis-elements in the promoter regions of target genes to control the expression of downstream genes and initiate a cascade of physiological and biochemical responses in plant cells (Ramirez and Basu, 2009). miR160 targeting ARF transcription factor in response to *Pseudoperonospora cubensis* infection in cucumbers was identified (Jin and Wu, 2015). In potatoes, novel regulatory mechanisms for JA- and SA-mediated crosstalk in defense responses were identified, with novel regulation of the SA pathway by JA through *StNPR1* (a defense gene) during infection with the potato necrotrophic pathogen. In parallel, the miR160 target gene *StARF16* (a gene involved in growth and development) regulates *StNPR1* gene expression and thereby inhibits the SA pathway (Kalsi et al., 2022). In *B. rapa*, miR319a is involved in regulating plant resistance to stem rot disease caused by *Sclerotinia sclerotiorum*, and overexpression of MIR319A reduced plant resistance to SSR due to interruption of the JA- and SA-related pathways (Dong et al., 2021). AP2 belongs to the AP2/EREBP family of transcription factors and plays an important role in pathogen resistance (Cheng et al., 2016). AP2 is involved in the biological stress response through the ethylene signaling pathway and protects plants from pathogen attack (Zimmerli et al., 2004). Liang et al. (2019) constructed a network of miRNAs and target genes associated with cucumber–CGMMV interactions and found that the target gene ethylene response transcription factor PAP2-7 of miR172 regulates downstream gene expression in response to pathogen defense. In tomatoes, overexpression of miR172a and miR172b increased resistance to *Phytophthora infestans* infection by suppressing the AP2/ERF transcription factor (Luan et al., 2018). A new microRNA, can-miRn37a, was identified in pepper that regulate resistance to anthracnose pathogen *Colletotrichum truncatum* L. by suppressing the expression of ethylene response factors (Mishra et al., 2018).

## MicroRNAs regulate the production of ROS

Reactive oxygen species (ROS) is an important defense response of plants against pathogenic infestation. Increasing evidence points to a potential role of microRNAs in oxidative stresses. In Arabidopsis, miR398 targets two closely related Cu/Zn superoxide dismutases that can detoxify superoxide radicals (Sunkar et al., 2006). In rice, miR528 negatively regulates viral resistance by cleaving *L*-*ascorbate oxidase* (AO) messenger RNA, thereby reducing AO-mediated accumulation of ROS (Wu et al., 2017). Similar results were reported in tomatoes. Hong et al. (2019) reported that transgenic tomato plants overexpressing miR482c had reduced *NBS*-*LRR* expression and reduced ROS scavenging capacity after late blight infection, and thus miR482 was a negative regulator of tomato resistance. Sly-miR397 affects the expression levels of ROS scavenging genes, altering H_2_O_2_ concentrations in response to *Phytophthora infestans* and *Oidium neolycopersici* infections (Guan et al., 2022). Sly-miR159 targets SlMyb33 transcription factor, and SlMYB33 promotes the expression of the resistance gene *SlSw5a*. Silencing neither *SlMyb33* nor *SlSw5a* leads to a decrease in the ROS level and confers *tomato leaf curl New Delhi virus* susceptibility (Sharma et al., 2021). miR6024 overexpression in tomato plants exhibits downregulation of the target gene *NLR*, excessive accumulation of ROS, and hypersusceptibility to *A. solani* infection; therefore, miR6024–NLR interactions negatively regulate *A. solani* pathogenesis in tomatoes (Dey et al., 2022).

## Discussion

The horticultural plants comprise a large collection of plants that significantly contribute to food, fuels, beauty of living places, and ecosystems. Horticultural plants are mainly cultivated in solar greenhouses and plastic greenhouses, with large temperature difference, high humidity, and a threat to serious diseases. The development of the horticultural industry is largely limited by disease and excessive pesticide application. The most effective strategies for preventing diseases in horticultural plants include selecting disease-resistant gene resources and breeding disease-resistant varieties.

In the past few years, microRNAs have emerged as important regulators of both growth and disease resistance in horticultural plants. Artificial targeting of microRNAs or targets is an attractive approach for improving disease resistance and for molecular plant breeding. There are two main challenges of microRNA research on horticultural plants. The first challenge is the elaboration of the disease resistance of certain microRNA. Understanding which microRNA works in regulating disease resistance is an important step. Although a great progress has been made in a few model species, the same has not been achieved in many other horticultural plants. The second challenge is the lack of an effective transformation system in many horticultural plants. The identified microRNAs need to be functionally validated in horticultural plants. Recently, a set of tobacco ringspot virus-based vectors was developed for studying microRNA function in cucurbits (Fang et al., 2021), which shed light on microRNA function in other horticultural plants. Therefore, we should accelerate the identification and functional analysis of key microRNAs involved in the disease resistance mechanism and try to apply them to disease resistance breeding.

## References

[B1] Afzal A. J. Wood A. J. Lightfoot D. A. (2008). Plant receptor-like serine threonine kinases: Roles in signaling and plant defense. Mol. Plant Microbe Interact. 21, 507–517. 10.1094/MPMI-21-5-0507 18393610

[B2] Bologna N. G. Voinnet O. (2014). The diversity, biogenesis, and activities of endogenous silencing small RNAs in Arabidopsis. Annu. Rev. Plant Biol. 65, 473–503. 10.1146/annurev-arplant-050213-035728 24579988

[B3] Borges F. Martienssen R. A. (2015). The expanding world of small RNAs in plants. Nat. Rev. Mol. Cell Biol. 16, 727–741. 10.1038/nrm4085 26530390 PMC4948178

[B4] Canto-Pastor A. Santos B. Valli A. A. Summers W. Schornack S. Baulcombe D. C. (2019). Enhanced resistance to bacterial and oomycete pathogens by short tandem target mimic RNAs in tomato. Proc. Natl. Acad. Sci. U. S. A. 116, 2755–2760. 10.1073/pnas.1814380116 30679269 PMC6377479

[B5] Chen C. Li J. Feng J. Liu B. Feng L. Yu X. (2021). sRNAanno-a database repository of uniformly annotated small RNAs in plants. Hortic. Res. 8, 45. 10.1038/s41438-021-00480-8 33642576 PMC7917102

[B6] Chen C. Zeng Z. Liu Z. Xia R. (2018). Small RNAs, emerging regulators critical for the development of horticultural traits. Hortic. Res. 5, 63. 10.1038/s41438-018-0072-8 30245834 PMC6139297

[B7] Cheng H. Y. Wang Y. Tao X. Fan Y. F. Dai Y. Yang H. (2016). Genomic profiling of exogenous abscisic acid-responsive microRNAs in tomato (*Solanum lycopersicum*). BMC Genomics 17, 423. 10.1186/s12864-016-2591-8 27260799 PMC4891822

[B8] Dey S. Sarkar A. Chowdhury S. Singh R. Mukherjee A. Ghosh Z. (2022). Heightened miR6024-NLR interactions facilitate necrotrophic pathogenesis in tomato. Plant Mol. Biol. 109, 717–739. 10.1007/s11103-022-01270-z 35499677

[B9] Dong W. Ren W. Wang X. Mao Y. He Y. (2021). MicroRNA319a regulates plant resistance to *Sclerotinia stem rot* . J. Exp. Bot. 72, 3540–3553. 10.1093/jxb/erab070 33606883

[B10] Dong Y. Zhu H. Qiu D. (2023). Hrip1 enhances tomato resistance to yellow leaf curl virus by manipulating the phenylpropanoid biosynthesis and plant hormone pathway. Biotech 13, 11. 10.1007/s13205-022-03426-6 PMC9755419 36532856

[B11] Fang L. Wei X. Liu L. Zhou L. Tian Y. Geng C. (2021). A tobacco ringspot virus-based vector system for gene and microRNA function studies in cucurbits. Plant Physiol. 186, 853–864. 10.1093/plphys/kiab146 33764466 PMC8195500

[B12] Feng J. Liu S. Wang M. Lang Q. Jin C. (2014). Identification of microRNAs and their targets in tomato infected with *Cucumber mosaic virus* based on deep sequencing. Planta 240, 1335–1352. 10.1007/s00425-014-2158-3 25204630

[B13] Guan Y. Wei Z. Song P. Zhou L. Hu H. Y. Hu P. (2022). MicroRNA expression profiles in response to Phytophthora infestans and Oidium neolycopersici and functional identification of sly-miR397 in tomato. Phytopathology. Accession Number: 36346372. 10.1094/PHYTO-04-22-0117-R 36346372

[B14] Hong Y. H. Meng J. He X. L. Zhang Y. Y. Luan Y. S. (2019). Overexpression of MiR482c in tomato induces enhanced susceptibility to late blight. Cells 8, 822. 10.3390/cells8080822 31382588 PMC6721620

[B15] Hong Y. Meng J. He X. Zhang Y. Liu Y. Zhang C. (2021). Editing miR482b and miR482c simultaneously by CRISPR/Cas9 enhanced tomato resistance to *Phytophthora infestans* . Phytopathology 111, 1008–1016. 10.1094/PHYTO-08-20-0360-R 33258411

[B16] Ji H. M. Zhao M. Gao Y. Cao X. X. Mao H. Y. Zhou Y. (2018). FRG3, a target of slmiR482e-3p, provides resistance against the fungal pathogen *Fusarium oxysporum* in tomato. Front. Plant Sci. 9, 26. 10.3389/fpls.2018.00026 29434609 PMC5797444

[B17] Jiang N. Cui J. Hou X. Yang G. Xiao Y. Han L. (2020). Sl-lncRNA15492 interacts with Sl-miR482a and affects *Solanum lycopersicum* immunity against *Phytophthora infestans* . Plant J. 103, 1561–1574. 10.1111/tpj.14847 32432801

[B18] Jiang N. Meng J. Cui J. Sun G. Luan Y. (2018). Function identification of miR482b, a negative regulator during tomato resistance to Phytophthora infestans. Hortic. Res. 5, 9. 10.1038/s41438-018-0017-2 29507733 PMC5830410

[B19] Jin W. Wu F. (2015). Identification and characterization of cucumber microRNAs in response to *Pseudoperonospora cubensis* infection. Gene 569, 225–232. 10.1016/j.gene.2015.05.064 26071186

[B20] Kalsi H. S. Karkhanis A. A. Natarajan B. Bhide A. J. Banerjee A. K. (2022). AUXIN RESPONSE FACTOR 16 (StARF16) regulates defense gene *StNPR1* upon infection with necrotrophic pathogen in potato. Plant Mol. Biol. 109, 13–28. 10.1007/s11103-022-01261-0 35380408

[B21] Liang C. Liu H. Hao J. Li J. Luo L. (2019). Expression profiling and regulatory network of cucumber microRNAs and their putative target genes in response to *cucumber green mottle mosaic virus* infection. Arch. Virol. 164, 1121–1134. 10.1007/s00705-019-04152-w 30799510 PMC6420491

[B22] Llave C. Xie Z. Kasschau K. D. Carrington J. C. (2002). Cleavage of Scarecrow-like mRNA targets directed by a class of Arabidopsis miRNA. Science 297, 2053–2056. 10.1126/science.1076311 12242443

[B23] Luan Y. Cui J. Li J. Jiang N. Liu P. Meng J. (2018). Effective enhancement of resistance to *Phytophthora infestans* by overexpression of miR172a and b in *Solanum lycopersicum* . Planta 247, 127–138. 10.1007/s00425-017-2773-x 28884358

[B24] Mishra R. Mohanty J. N. Chand S. K. Joshi R. K. (2018). Can-miRn37a mediated suppression of ethylene response factors enhances the resistance of chilli against anthracnose pathogen *Colletotrichum truncatum* L. Plant Sci. 267, 135–147. 10.1016/j.plantsci.2017.12.001 29362092

[B25] Modepalli V. Fridrich A. Agron M. Moran Y. (2018). The methyltransferase HEN1 is required in *Nematostella vectensis* for microRNA and piRNA stability as well as larval metamorphosis. PLoS Genet. 14 (8), e1007590. 10.1371/journal.pgen.1007590 30118479 PMC6114907

[B26] Ouyang S. Park G. Atamian H. S. Han C. S. Stajich J. E. Kaloshian I. (2014). MicroRNAs suppress NB domain genes in tomato that confer resistance to *Fusarium oxysporum* . PLoS Pathog. 10, e1004464. 10.1371/journal.ppat.1004464 25330340 PMC4199772

[B27] Park M. Y. Wu G. Gonzalez-Sulser A. Vaucheret H. Poethig R. S. (2005). Nuclear processing and export of microRNAs in Arabidopsis. Proc. Natl. Acad. Sci. U. S. A. 102 (10), 3691–3696. 10.1073/pnas.0405570102 15738428 PMC553294

[B28] Paul P. Chhapekar S. S. Rameneni J. J. Oh S. H. Dhandapani V. Subburaj S. (2021). MiR1885 regulates disease tolerance genes in *Brassica rapa* during early infection with *Plasmodiophora brassicae* . Int. J. Mol. Sci. 22, 9433. 10.3390/ijms22179433 34502341 PMC8430504

[B29] Ramirez S. R. Basu C. (2009). Comparative analyses of plant transcription factor databases. Curr. Genomics 10, 10–17. 10.2174/138920209787581253 19721806 PMC2699837

[B30] Sharma N. Muthamilarasan M. Dulani P. Prasad M. (2021). Genomic dissection of ROS detoxifying enzyme encoding genes for their role in antioxidative defense mechanism against *Tomato leaf curl New Delhi virus* infection in tomato. Genomics 113, 889–899. 10.1016/j.ygeno.2021.01.022 33524498

[B31] Shivaprasad P. V. Chen H. M. Patel K. Bond D. M. Santos B. A. Baulcombe D. C. (2012). A microRNA superfamily regulates nucleotide binding site-leucine-rich repeats and other mRNAs. Plant Cell 24 (3), 859–874. 10.1105/tpc.111.095380 22408077 PMC3336131

[B32] Sunkar R. Kapoor A. Zhu J. K. (2006). Posttranscriptional induction of two Cu/Zn superoxide dismutase genes in Arabidopsis is mediated by downregulation of miR398 and important for oxidative stress tolerance. Plant Cell 18, 2051–2065. 10.1105/tpc.106.041673 16861386 PMC1533975

[B33] Voinnet O. (2009). Origin, biogenesis, and activity of plant microRNAs. Cell 136, 669–687. 10.1016/j.cell.2009.01.046 19239888

[B34] Wu J. G. Yang R. X. Yang Z. R. Yao S. Z. Zhao S. S. Wang Y. (2017). ROS accumulation and antiviral defence control by microRNA528 in rice. Nat. Plants 3, 16203. 10.1038/nplants.2016.203 28059073

[B35] Yu X. Y. Hou Y. J. Cao L. F. Zhou T. T. Wang S. H. Hu K. X. (2022). MicroRNA candidate miRcand137 in apple is induced by Botryosphaeria dothidea for impairing host defense. Plant Physiol. 189, 1814–1832. 10.1093/plphys/kiac171 35512059 PMC9237668

[B36] Yu Y. Jia T. Chen X. (2017). The 'how' and 'where' of plant microRNAs. New Phytol. 216, 1002–1017. 10.1111/nph.14834 29048752 PMC6040672

[B37] Zhai J. Jeong D. H. De Paoli E. Park S. Rosen B. D. Li Y. (2011). MicroRNAs as master regulators of the plant NB-LRR defense gene family via the production of phased, trans-acting siRNAs. Genes & Dev. 25 (23), 2540–2553. 10.1101/gad.177527.111 22156213 PMC3243063

[B38] Zhang R. Zheng F. Wei S. Zhang S. Li G. Cao P. (2019). Evolution of disease defense genes and their regulators in plants. Int. J. Mol. Sci. 20, 335. 10.3390/ijms20020335 30650550 PMC6358896

[B39] Zimmerli L. Stein M. Lipka V. Schulze-Lefert P. Somerville S. (2004). Host and non-host pathogens elicit different jasmonate/ethylene responses in Arabidopsis. Plant J. 40, 633–646. 10.1111/j.1365-313X.2004.02236.x 15546348

